# Cost-Effectiveness of School Urinary Screening for Early Detection of IgA Nephropathy in Japan

**DOI:** 10.1001/jamanetworkopen.2023.56412

**Published:** 2024-02-16

**Authors:** Kimiko Honda, Yoko Akune, Rei Goto

**Affiliations:** 1 Center of Health Economics and Health Technology Assessment, Keio University Global Research Institute, Tokyo, Japan; 2 Graduate School of Health Management, Keio University, Tokyo, Japan; 3 Graduate School of Business Administration, Keio University, Tokyo, Japan

## Abstract

**Importance:**

The evidence for and against screening for chronic kidney disease in youths who are asymptomatic is inconsistent worldwide. Japan has been conducting urinary screening in students for 50 years, allowing for a full economic evaluation that includes the clinical benefits of early detection and intervention for chronic kidney disease.

**Objectives:**

To evaluate the clinical effectiveness and cost-effectiveness of school urinary screening in Japan, with a focus on the benefits of the early detection and intervention for IgA nephropathy, and to explore key points in the model that are associated with the cost-effectiveness of the school urinary screening program.

**Design, Setting, and Participants:**

This economic evaluation with a cost-effectiveness analysis used a computer-simulated Markov model from the health care payer’s perspective among a hypothetical cohort of 1 000 000 youths aged 6 years in first grade in Japanese elementary schools, followed up through junior and high school. The time horizon was lifetime. Costs and clinical outcomes were discounted at a rate of 2% per year. Costs were calculated in Japanese yen and 2020 US dollars (¥107 = US $1).

**Interventions:**

School urinary screening for IgA nephropathy was compared with no screening.

**Main Outcomes and Measures:**

Outcomes were costs and quality-adjusted life-years (QALYs). Cost-effectiveness was determined by evaluating whether the incremental cost-effectiveness ratio (ICER) per QALY gained remained less than ¥7 500 000 (US $70 093).

**Results:**

In the base case analysis, the ICER was ¥4 186 642 (US $39 127)/QALY, which was less than the threshold. There were 60.3 patients/1 000 000 patients in the no-screening strategy and 31.7 patients/1 000 000 patients in the screening strategy with an end-stage kidney disease. Cost-effectiveness improved as the number of screenings decreased (screening frequency <3 times: incremental cost, −¥75 [US $0.7]; incremental QALY, 0.00025; ICER, dominant), but the number of patients with end-stage kidney disease due to IgA nephropathy increased (40.9 patients/1 000 000 patients). Assuming the disutility due to false positives had a significant impact on the analysis; assuming a disutility of 0.01 or more, the population with no IgA nephropathy had an ICER greater than the threshold (¥8 304 093 [US $77 608]/QALY).

**Conclusions and Relevance:**

This study found that Japanese school urinary screening was cost-effective, suggesting that it may be worthy of resource allocation. Key factors associated with cost-effectiveness were screening cost, the probability of incident detection outside of screening, and IgA nephropathy incidence, which may provide clues to decision-makers in other countries when evaluating the program in their own context.

## Key Points

**Question:**

Is the nationwide urinary screening program for students in Japan cost-effective regarding early detection and intervention of IgA nephropathy?

**Findings:**

In this economic evaluation of a hypothetical 1 000 000 children aged 6 years, the school urinary screening strategy cost was ¥4 186 642 (US $39 127) per quality-adjusted life-year gained compared with the no screening strategy, and the number of patients with end-stage kidney failure due to IgA nephropathy was reduced from 60.3 to 31.7 students/1 000 000 individuals.

**Meaning:**

This study found that the school urinary screening program in Japan was cost-effective but cost-effectiveness depended on screening costs, annual probability of incident detection outside screening, and IgA nephropathy incidence.

## Teaser

This economic evaluation examines the cost-effectiveness of a urinary screening programs for early detection of IgA nephropathy among students in Japan.

## Introduction

Chronic kidney disease (CKD) screening in youths is a controversial topic.^1^ In Japan, Korea, and Taiwan, the urinary screening of students is mandatory for early detection of primary glomerulonephritis, including IgA nephropathy (IgAN). However, the frequency of inaccurate results has led to questions in other countries about the cost-effectiveness of CKD screening in youths who are asymptomatic.^2^ In Japan, school urinary screening for youths has been mandatory since 1973, showing effectiveness in reducing end-stage kidney disease (ESKD) due to primary glomerulonephritis in individuals younger than 20 years.^3,4,5,6^ Notwithstanding, to our knowledge, the economic evaluation of school urinary screening has never been conducted in Japan or other countries with school urinary screening.

To our knowledge, only 2 economic evaluations of urinary screening for youths who are asymptomatic exist, namely a cost analysis for urinary screening in youths who were asymptomatic by Kaplan et al^7^ and a cost-effectiveness analysis with CKD detection as an outcome by Sekhar et al.^8^ They raised concerns about the high rate of false-positive and transient abnormalities and concluded that the screening was not cost-effective. Notably, neither study evaluated the clinical effectiveness of CKD’s early detection and treatment. CKD can significantly damage quality of life and social outcomes of youths throughout life.^9,10,11^ If CKD progresses to dialysis, the annual cost per patient is estimated at $66 000 in the US and ¥5 000 000 (US $46 729) in Japan.^12,13^ To assess the value of the urinary screening program, a full economic evaluation that includes the effectiveness of CKD early detection and intervention is needed.

This study aimed to first assess the cost-effectiveness of school urinary screening in Japan, focusing on the effectiveness of early detection of IgAN, the screening’s primary target. Second, we aimed to explore key points associated with the school urinary screening’s cost-effectiveness.

## Methods

This economic evaluation followed the Consolidated Health Economic Evaluation Reporting Standards (CHEERS) reporting guideline. This study was based on published reports and did not involve human participants; therefore, institutional review board approval and informed consent under 45 CFR §46 were not sought.

### School Urinary Screening in Japan and IgAN

In Japan, annual urinalysis is mandatory for all students, from kindergarten to university; local governments oversee implementation. The screening program is described in depth elsewhere.^5,6^ In elementary and junior high schools, the screening rate is almost 100%.^14^ Urine samples are collected at home in the early morning and tested for hematuria and proteinuria using urine dipsticks; individuals who test positive are retested. Students testing positive 2 consecutive times are subjected to a full medical examination. Screening costs are fully covered by public funds, but the cost of a full examination may be covered by public funds or insurance, depending on the local government. Matsumura et al^14^ reported that among 9544 students subjected to the full medical examination in Chiba City, there were 204 individuals with IgAN, 54 individuals with non-IgA chronic nephritis, 22 individuals with membranous proliferative glomerulonephritis, and 15 individuals with membranous nephropathy.

IgAN is the most common form of chronic glomerulonephritis. The incidence is higher in Asia, particularly Japan, with 4.5 to 9.9 diagnoses/100 000 students/y, and lower in the US (0.3-1.0 diagnoses/100 000 students/y) and Europe (0.2 diagnoses/100 000 students/y).^15,16,17,18,19^ This difference may be associated with genetic factors and environmental influences and the presence or absence of urinary screening programs and indications for kidney biopsy.^20^ The main symptoms of IgAN are microscopic hematuria and proteinuria. In Japan, 70% to 80% of IgAN cases in pediatric patients are detected in school urinalysis and 15% to 20% by macroscopic hematuria.^21^ Although kidney dysfunctions progress slowly, 20% to 30% of patients may develop severe CKD stage or ESKD within 20 years of long-term follow-up, and early diagnosis and treatment may improve adult prognosis.^22^

### Overview

To analyze the cost-effectiveness of school urinary screening for IgAN over 12 years in Japanese elementary, junior high, and high schools, we used a computer-simulated Markov model to compare costs and outcomes associated with the screening and those of the no-screening strategy. The model was evaluated using the expected value calculation (cohort analysis) with TreeAge Pro decision analysis software version 2021 (TreeAge Software LLC). A hypothetical cohort of 1 000 000 youths aged 6 years in first grade in Japanese schools was followed up until each person died or reached age 120 years to evaluate long-term screening effectiveness. The cycle duration was 1 year. We considered the public health care payer’s perspective regarding factors, such as costs, comparators, and target populations, within the scope of Japanese public health insurance and public health care. Outcomes were measured using quality-adjusted life-years (QALYs). A QALY value is calculated by multiplying life-years by a utility value determined by a preference-based measure. Based on the latest guidelines for economic evaluation in Japan, costs and outcomes were discounted at a rate of 2% per year.^23^ Costs were calculated in Japanese yen and 2020 US dollars (¥107 = US $1).

Results are presented as incremental cost-effectiveness ratios (ICERs) per QALY gained. An ICER of less than ¥7 500 000 (US $70 093) per QALY gained was considered cost-effective because this threshold is used for pediatric diseases in Japanese official health technology assessments. In this analysis, only childhood-onset IgAN (pediatric IgAN) and asymptomatic hematuria (ASH) were included; namely, we did not assess screening effectiveness or costs for other kidney diseases or for IgAN developing after age 18 years. ASH was defined by microscopic hematuria without hypertension, severe proteinuria, or kidney dysfunction detected incidentally during screening and not progressing to kidney disease. ASH was assessed to account for the association of screening with negative outcomes given that its detection brings no benefits and increases costs.

### Statistical Analysis

**Figure 1.**
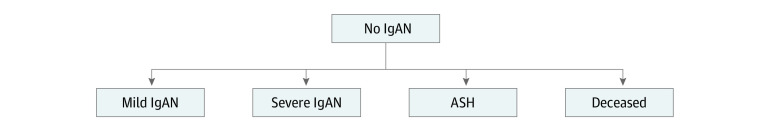
State Transition Diagram of Initial States at Cycle 0 ASH indicates asymptomatic hematuria; IgAN, IgA nephropathy.

The entire cohort in the no IgAN state at the beginning of the model was transitioned to 1 of 5 possible states: no IgAN, mild IgAN, severe IgAN, ASH, and deceased with initial probabilities (Figure 1). The population that stayed in the no IgAN state had the same probability of transitioning to these 5 states at the next cycle and afterward. Mild and severe IgAN were defined according to the Japanese Society for Pediatric Nephrology (eAppendix 1 in Supplement 1).^24^ IgAN or ASH could be detected at a screening (only in the screening strategy), detected at an incidental visit, or remain undetected (eFigures 1-3 and eAppendix 3 in Supplement 1). Patients with IgAN or ASH followed the same course after diagnosis in both strategies. All patients with IgAN diagnosed by kidney biopsy started treatment (eAppendix 2 in Supplement 1). Patients who progressed to ESKD underwent peritoneal dialysis, hemodialysis, or kidney transplant (eFigure 4 in Supplement 1). In both strategies, populations followed the same path after age 18 years. Patients with IgAN not detected by age 18 years were combined into a single state at age 18 years and could be detected through general resident or workplace health checkups or incidental visits. Patients with IgAN detected after ager 17 years were reassigned according to ESKD progression risk (eFigure 5 in Supplement 1).

Branching progressions occurred from 5 states: no IgAN, mild or severe IgAN, ASH, and undetected IgAN before age 18 years (eFigures 6-10 and eAppendix 4 in Supplement 1). The validity of the model concept, input data, and outcomes was confirmed by a pediatric nephrologist (K.H.) and a nephrologist. The model was also validated by comparison with the actual number of patients aged 18 years or younger with ESKD due to IgAN. Estimates of the actual number of patients were based on 2 Japanese Society for Pediatric Nephrology surveys.^3,25^

#### Probabilities

To be the most conservative for the screening strategy, we set the IgAN incidence as the lowest value of previous reports in Japan.^15,16,17^ In several reports of patients with severe and mild IgAN (ie, diagnosed on kidney biopsy), severe case proportions were as high as 40%.^15,26,27,28^ However, given the presence of mild IgAN for which no kidney biopsy was performed, the proportion of patients with severe IgAN should be even lower. The annual probability of incidental visits of patients with IgAN was assumed to be 0.22 based on the proportion of patients detected outside of screening during the year.^15,16^ Therefore, if patients not detected in that 1 year were included in the denominator, the annual probability of incidental visits of patients with IgAN would be lower than 0.22. Estimating a high probability of incidental visits of patients with IgAN leads to conservative results. Given that serious complications of kidney biopsy that could permanently affect kidney function in Japan are rare, complications from kidney biopsy were not considered in the model.^29^

Findings from randomized clinical trials (RCTs) in Japan were used to determine IgAN treatment outcomes.^30,31^ No serious side effects were reported in RCTs of 2-year angiotensin-converting enzyme inhibitor treatment in mild IgAN and 2-year combination treatment in severe IgAN; thus, treatment side effects and dropouts were not considered.

#### Costs and Utilities

The per-patient screening cost was calculated by summing the cost of urine containers, carriage, dipstick, and miscellaneous costs from the literature.^32^ Costs of detailed examination, treatment, and outpatient follow-up visits were estimated according to the national medical care fee schedule and expert committee and expert opinion recommendations.^24,33,34^ Annual medical costs per patient with dialysis or transplant were obtained from previous studies.^13^

Utility values were set at 1.0 for individuals in the no IgAN, ASH, and IgAN states without dialysis or transplant. Although there have been no reports of disutility due to IgAN, we accounted for disutility during treatment in sensitivity analysis. However, no data were available for youths with dialysis or transplant in Japan; therefore, values from Australian and New Zealand youths and Japanese adults with dialysis or transplant were used.^35,36,37^

#### Sensitivity and Scenario Analyses

We performed sensitivity analysis to capture parameter uncertainty. In the 1-way sensitivity analysis, costs and probabilities varied by 50% increases and decreases and utilities varied by 20% increases and decreases. As an exception, the incidence of IgAN varied among previously reported values. The sensitivity of screening for mild IgAN and ASH varied between 0.6 and 1.0. Remission rates after 2 years of angiotensin-converting enzyme inhibitor treatment for mild IgAN and combination treatment for severe IgAN were based on RCT results.^30,31^ Therefore, the variability range was reduced to increases and decreases of 20%. Although the probability of progression to ESKD among patients with mild IgAN with residual proteinuria after treatment was set at 0 in the base case, the upper limit in the sensitivity analysis was set to the value in untreated mild IgAN. We also conducted a probabilistic sensitivity analysis in which model inputs were simultaneously varied over plausible distributions. The β distribution was applied for probabilities and utilities, and the γ distribution was used for costs.

Scenario analysis was performed to identify the impact of reducing the number of screenings over the 12-year period and impact of assumption of disutility due to false positives in the screening. The number of screenings was reduced by raising the screening starting age by 1 year from age 10 years in scenario 1 and reducing the frequency of screening in scenario 2. In scenario 3, the presence of disutility due to false positives in the screening in the no IgAN population was assumed. We found no reports on the disutility due to false positives in CKD screening. Thus, referring to a previous review of cancer screening,^38^ we assumed that the no IgAN population would experience a utility loss of 0.05 to 0.20 for 2 weeks when diagnosed as positive at screening (2 consecutive positive urinalysis results).

## Results

### Parameters

Table 1 lists main input parameters identified by this model. Other parameters are listed in eTables 1 to 3 and eAppendix 5 in Supplement 1.^14,15,16,17,26,27,28,30,31,32,35,36,37,39,40^ The annual incidence of IgAN was set at 4.5 cases per 100 000 people. The sensitivity of the screening for mild IgAN was assumed to be 0.8, referencing a previous study^16^ in Niigata City, Japan. The cost of screening per person was ¥200 (US $1.9). The utility was assumed to be 1.0 whether or not IgAN was present, but after the introduction of kidney replacement therapy, it decreased to 0.61 for individuals younger than age 20 years, 0.75 for those aged 20 years and older, 0.76 after kidney transplantation for those younger than 20 years, and 0.89 after kidney transplantation for those aged 20 years old and older (Table 1).

**Table 1.** Main Input Parameters

Parameter	Value	Low	High	Source
Probability				
Annual incidence of IgAN	0.000045	0.000045	0.000099	Utsunomiya et al,^15^ 2003; Ikezumi et al,^16^ 2008; Shibano et al,^17^ 2016
Proportion of severe IgAN	0.3	−50%	+50%	Utsunomiya et al,^15^ 2003; Yoshikawa et al,^26^ 1992; Yata et al,^27^ 2008;^-^Higa et al,^28^ 2015
Annual probability of progression to ESKD without treatment				
Patients with mild IgAN	0.0016	−50%	+50%	Yata et al,^27^ 2008; Higa et al,^28^ 2015
Patients with severe IgAN	0.029	−50%	+50%	Yata et al,^27^ 2008; Kamei et al,^39^ 2011
Proportion with remission at end of treatment				
Patients with mild IgAN	0.89	−20%	+20%	Shima et al,^30^ 2019
Patients with severe IgAN	0.76	−20%	+20%	Shima et al,^31^ 2018
School urinary screening rate	0.98	NA^a^	NA^a^	Matsumura,^14^ 2013
Health checkup rate (≥19 y)	0.7	−50%	+50%	Ministry of Health, Labour and Welfare of Japan,^40^ 2019
Probability of receiving full medical examination (<18 y)	0.7	−50%	1.0	Matsumura,^14^ 2013; Utsunomiya et al,^15^ 2003
False-positive rate for school urinary screening	0.0047	−50%	+50%	Utsunomiya et al,^15^ 2003; Ikezumi et al,^16^ 2008; Shibano et al,^17^ 2016
Sensitivity of school urinary screening for mild IgAN	0.8	0.6	1.0	Author assumption
Cost, ¥				
Screening	200	100	300	Murakami et al,^32^ 2003^b^
Detailed examination	18 940	−50%	+50%	National fees and expert recommendations^c^
Health state				
Healthy	1.0	NA^a^	NA^a^	NA^a^
IgAN treatment	1.0	0.9	1.0	Author assumption
PD or HD (<20 y)	0.61	−20%	+20%	Francis et al,^35^ 2019
PD or HD (≥20 y)	0.75	−20%	+20%	Noto et al,^36^ 2021
KT (<20 y)	0.76	−20%	+20%	Francis et al,^35^ 2019
KT (≥20 y)	0.89	0.71	1.0	Hiragi et al,^37^ 2019

Abbreviations: ESKD, end-stage kidney disease; HD, hemodialysis; IgAN, IgA nephropathy; KT, kidney transplant; NA, not applicable; PD, peritoneal dialysis.

Currency conversion: To convert yen to 2020 US dollars, divide by 107.

^a^ Sensitivity analysis was not performed.

^b^ Murakami et al^32^ calculated the cost per specimen as ¥150 for the urine collection container and collection costs, ¥30 for the urine test strips, and ¥20 for other miscellaneous costs, for a total of ¥200. This is generally at public expense (eAppendix 1 in Supplement 1).

^c^ Set according to the national medical care fee schedule and recommendations of an expert committee and expert opinion.

### Model Validity

A Japanese Society of Pediatric Nephrology nationwide survey reported the per-year incidence of ESKD (2006-2011) as 4.0 cases/1 000 000 individuals in the age-related population,^25^ 5.9% of which were due to glomerulonephritis. In 2002, IgAN accounted for 37% of glomerulonephritis cases that caused ESKD.^3^ Accordingly, the annual ESKD incidence due to IgAN was estimated to be 0.087 cases/1 000 000 individuals. By contrast, the screening strategy in the model showed that ESKD incidence due to IgAN from age 6 to 19 years was 0.0254 cases/1 000 000 people/y. The difference in these incidence rates is 0.06258 cases/1 000 000 people/y, and the difference is less than 1 person/y after 12 years of follow-up of a hypothetical cohort of 1 000 000 people. Therefore, this difference was considered acceptable for the analysis.

### Summary of Results

Table 2 summarizes base case and scenario analyses. All results of scenario analysis are in eTables 4 and 5 and eAppendix 6 in Supplement 1. In the base case analysis, the cost was ¥8338 (US $78) and the QALY was 39.45001 for the nonscreening strategy; these figures were ¥9943 (US $93) and 39.45039, respectively, for the screening strategy. The incremental cost and incremental QALY of the screening vs nonscreening strategy were ¥1605 (US $15) and 0.00038, respectively. Hence, the ICER was ¥4 186 642 (US $39 127)/QALY. The number of patients with ESKD due to IgAN per 1 000 000 patients in the time horizon was 60.3 patients in the no-screening strategy and 31.7 patients in the screening strategy. The number of patients with IgAN undetected before age 18 years per 1 000 000 patients was 135.5 patients in the no-screening strategy and 27.3 patients in the screening strategy.

**Table 2.** Base Case and Scenario Cost-Effectiveness Analysis

Strategy	Cost, ¥	Incremental cost, ¥	QALY	Incremental QALY	ICER, ¥/QALY	Patients, No./1 000 000 individuals	
						IgAN undetected before age 18 y^a^	ESKD lifetime^b^
Base case							
No screening	8338	NA	39.45001	NA	NA	135.5	60.3
Screening	9943	1605	39.45039	0.00038	4 186 642	27.3	31.7
Scenario 1: varying starting age of screening, y							
10	9078	740	39.45036	0.00035	2 114 612	27.4	33.8
15	8223	−115	39.45027	0.00027	Dominant	33	39.4
Scenario 2: varying screening frequency							
Every 2 y (starting at age 7 y)	8761	423	39.45032	0.00032	1 337 699	41.4	36.6
Every 3 y (starting at age 8 y)	8431	93	39.45028	0.00027	338 734	49.7	39.6
3 Times (ages 11, 14, and 17 y)	8263	−75	39.45026	0.00025	Dominant	50.1	40.9
Scenario 3: disutility due to false positive							
0.05	9943^c^	1605^c^	39.45029	0.00029	5 566 729	27.3^c^	31.7^c^
0.10	9943^c^	1605^c^	39.45020	0.00019	8 304 093	27.3^c^	31.7^c^
0.15	9943^c^	1605^c^	39.45010	0.00010	16 338 170	27.3^c^	31.7^c^
0.20	9943^c^	1605^c^	39.45001	0.00003	502 464 161	27.3^c^	31.7^c^

Abbreviations: ESKD, end-stage kidney disease; ICER, incremental cost-effectiveness ratios; IgAN, IgA nephropathy; NA, not applicable; QALY, quality-adjusted life-years.

Currency conversion: To convert yen to 2020 US dollars, divide by 107.

^a^ Number of patients per 1 000 000 patients.

^b^ Cumulative number of patients per 1 000 000 patients over the time horizon.

^c^ Results were the same as results of base case analysis.

In scenario analysis, the screening strategy became dominant by increasing the age at screening initiation to 15 years or older in scenario 1 (incremental cost, −¥115 [US $1.1]; incremental QALY, 0.00027; ICER, dominant) and decreasing the screening frequency to fewer than 3 times in scenario 2 (incremental cost, −¥75 [US $0.7]; incremental QALY, 0.00025; ICER, dominant). However, the number of patients with ESKD or IgAN undetected before age 18 years increased. For example, with fewer than 3 screenings, there were 40.9 and 50.1 patients per 1 000 000 patients with ESKD or IgAN undetected before age 18 years, respectively. In scenario 3, assuming a disutility of at least 0.1 (0.0038 for 2 weeks) due to false positives in the no IgAN population, the ICER was above the threshold (¥8 304 093 [US $77 608]/QALY) (Table 2).

### Sensitivity Analysis

**Figure 2.**
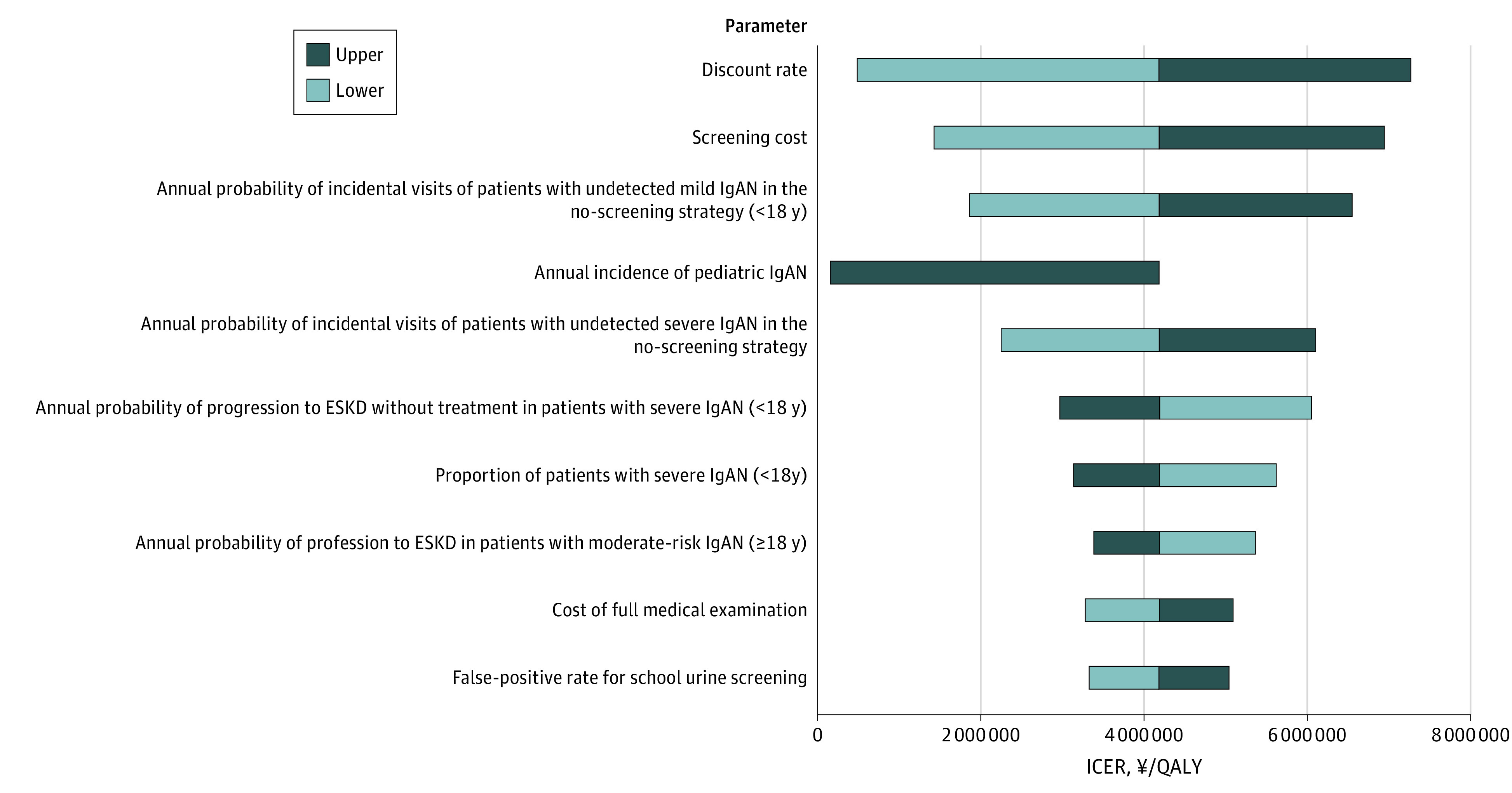
Tornado Diagram of 1-Way Sensitivity Analysis in Base-Case Analysis Ten parameters were selected in descending order of variation. Light portions of bars indicate the range of change when variables are set lower than values used in the base-case analysis, while dark portions indicate the range of change for variables set higher than base-case values. To convert yen to 2020 US dollars, divide by 107. ESKD indicates end-stage kidney disease; ICER, incremental cost-effectiveness ratio; IgAN, IgA nephropathy; QALY, quality-adjusted life-year.

In 1-way sensitivity analysis, primary model drivers were the discount rate, screening cost, annual probability of incidental visits for mild and severe IgAN, and annual incidence of pediatric IgAN (Figure 2). ICERs did not exceed the threshold of ¥7 500 000 ($70 093)/QALY for changes in the variables over the specified range. The probabilistic sensitivity analysis demonstrated that in 6620 of 10 000 simulations (66.2%), screening was cost-effective at the threshold (eFigure 11 and eAppendix 7 in Supplement 1).

## Discussion

In this economic evaluation with cost-effectiveness analysis, 12 years of a school urinary screening program in Japan was associated with a reduced number of patients with ESKD and was cost-effective. The screening cost, annual probability of incidental visits of patients with IgAN, and incidence of IgAN were key to the screening program’s cost-effectiveness. The assumption of disutility due to false positives significantly influenced results. To our knowledge, this is the first economic evaluation of urinary screening in youths who were asymptomatic that incorporates the clinical effectiveness of CKD early detection in the model.

The cost-effectiveness of CKD screening for youths remains questionable owing to the low prevalence of CKD and the frequent occurrence of inaccurate results given that even countries that have the screening program have not provided answers.^1^ To address this long-standing question, we focused on the clinical benefit associated with early treatment of IgAN (ie, screening’s primary target) and developed a precise model that replicates the epidemiology of IgAN in Japan, the treatment approach according to Japanese guidelines, and the outcomes associated with treatment among Japanese patients. The model provides a reliable framework for assessing the cost-effectiveness of school urinary screening in Japan. Our findings suggest that the screening program’s associated benefits justify its costs and support the allocation of health care resources to this Japanese program.

However, as with most economic evaluations, results cannot be directly applied elsewhere. This is despite comparable outcomes that could be achieved in Korea and Taiwan given that both countries already have screening programs in place and an IgAN incidence similar to that of Japan. Moreover, the incidence of IgAN varies significantly by country.^19,41^ Even if we allow for the possibility of underestimation owing to the lack of screening and differences in biopsy selection practices, incidence rates outside Asia, particularly in the US and Europe, may be considerably lower than rates used in this study. Additionally, when urinary screening is introduced in a country, costs initially increase due to costs associated with building a new system; these costs were not included in this analysis. These differences lead to poor cost-effectiveness. However, IgAN is already advanced at the time of diagnosis in countries that do not have such screening.^41^ This may mean that the probability of incidental visits by patients with undiagnosed IgAN is low. In these countries, the early detection of IgAN by screening may provide considerably greater benefits than assumed in this analysis. Therefore, decision-makers must evaluate the program’s cost-effectiveness in their countries based on these considerations. However, even if the program is considered less cost-effective, modifications to the screening protocol may bring the ICER within the threshold. In this study, annual screening as currently implemented was most effective within the ICER threshold. However, our findings suggest that in settings with different conditions or limited financial resources, a protocol with an increased screening starting age may be optimal given that the mean age of onset of pediatric IgAN is approximately 10 years.^42^ When each country adopts a screening program, the optimal screening protocol depends on the willingness to pay for the screening. In addition, several novel biomarkers associated with IgAN have been proposed in recent years.^43^ If these are sufficiently accurate and inexpensive to use for screening, the cost-effectiveness of screening programs could be further improved.

Importantly, the impact of disutility (ie, the cost of multiple testing resulting from false positive or transient abnormality in the base case analysis and the psychological burden) was examined in our scenario analysis. Although the high rates of false positive or transient abnormality in urinary screening for youths have been highly concerning,^2,7,8^ no study, to our knowledge, had quantified the impact of this rate in the evaluation of CKD screenings. Our results demonstrate that if even a small amount of disutility would result, the impact on cost-effectiveness would not be negligible. The false positive rate is high even with excellent sensitivity and specificity for screening for low prevalence diseases.^44^ As with cancer screening, harms associated with CKD screening need further study.

### Limitations

This study has several limitations. First, we assessed only IgAN as a screening target. If other glomerulonephritis types that would benefit from screening are included, the cost-effectiveness may improve; cost-effectiveness may also be exacerbated by including other diseases that are likely to be detected by symptoms without screening or for which no treatment has been established to significantly improve prognosis. However, given the low detection frequency of these other diseases compared with IgAN, the impact will likely be small. Second, we assumed that the natural prognosis and treatment outcome would worsen if pediatric patients with IgAN remained undetected into adulthood. This was based on evidence that the prognosis for adult IgAN is worse than that for pediatric IgAN, and a delay in treatment initiation worsens kidney prognosis by causing irreversible pathological changes.^45,46^ However, if pediatric IgAN differs in nature from adult IgAN and has a better prognosis than adult-onset IgAN over time, this analysis overestimates the prognostic impact of delayed detection and may be even less cost-effective. Third, this analysis was conducted from the public payer’s perspective, and family productivity losses and quality of life were not assessed owing to data scarcity in Japan. Public health economic evaluations should preferably incorporate a societal perspective to capture broader individual and spillover outcomes.^47^ Although cost-effectiveness may be better in a cost-utility analysis of pediatric care when conducted from a social perspective,^48^ the burden on parents to attend medical visits associated with false positives may have a negative impact in this analysis. Fourth, costs of screening and detailed examination, which greatly affect the cost-effectiveness of screening, were based on assumptions. A cost-effectiveness analysis based on observed data is necessary, and this is a challenge for the future.

## Conclusions

In this economic evaluation with a cost-effectiveness analysis, we found that the Japanese 12-year school urinary screening program was effective, with an associated reduction in the number of patients with ESKD, and cost-effective, focusing on the early detection and treatment of IgAN. Screening costs, annual probability of incident detection outside screening, and IgAN incidence were key factors associated with the program’s cost-effectiveness.

## Notes

Supplement 1. **eAppendix 1.** Definitions of Mild and Severe IgA Nephropathy **eAppendix 2.** Treatment of IgA Nephropathy **eAppendix 3.** State Transition Diagram of Mild and Severe IgA Nephropathy, Asymptomatic Hematuria, End-Stage Kidney Disease, and Undetected IgA Nephropathy at Age 18 y **eFigure 1.** State Transition Diagram of Mild IgA Nephropathy **eFigure 2.** State Transition Diagram of Severe IgA Nephropathy **eFigure 3.** State Transition Diagram of Asymptomatic Hematuria **eFigure 4.** State Transition Diagram of End-Stage Kidney Disease **eFigure 5.** State Transition Diagram of Undetected IgA Nephropathy at Age 18 y **eAppendix 4.** Trees From States of Non IgA Nephropathy, Mild or Severe IgA Nephropathy, Asymptomatic Hematuria, and IgA Nephropathy Undetected Before Age 18 y in Screening Strategy **eFigure 6.** Tree From No IgA Nephropathy State in Screening Strategy **eFigure 7.** Tree From Mild IgA Nephropathy State in Screening Strategy **eFigure 8.** Tree From Severe IgA Nephropathy State in Screening Strategy **eFigure 9.** Tree of Asymptomatic Hematuria State in Screening Strategy **eFigure 10.** Tree of Undetected IgA Nephropathy Before Age 18 y State in Screening Strategy **eAppendix 5.** Parameters **eTable 1.** Model Probability Parameters of Cost Effectiveness Analysis for School Urinary Screening **eTable 2.** Costs of Cost Effectiveness Analysis for School Urinary Screening **eTable 3.** Utility of Cost Effectiveness Analysis for School Urinary Screening **eAppendix 6.** Results of Scenario Analysis for Cost-Effectiveness of School Urinary Screening **eTable 4.** Analysis Results for Scenario 1 of Cost Effectiveness Analysis for School Urinary Screening **eTable 5.** Analysis Results for Scenario 2 of Cost Effectiveness Analysis for School Urinary Screening **eAppendix 7.** Probabilistic Sensitivity Analysis **eFigure 11.** Incremental Cost-Effectiveness Scatterplot
eReferences.

Supplement 2.
Data Sharing Statement
