# Supplementary material for: Cost-Effectiveness of School Urinary Screening for Early Detection of IgA Nephropathy in Japan
Source: JAMA Netw Open. 2024 Feb 16;7(2):e2356412. doi: 10.1001/jamanetworkopen.2023.56412 (PMC10873767; doi:10.1001/jamanetworkopen.2023.56412)
Supplement: Supplement 1. — eAppendix 1. Definitions of Mild and Severe IgA Nephropathy eAppendix 2. Treatment of IgA Nephropathy eAppendix 3. State Transition Diagram of Mild and Severe IgA Nephropathy, Asymptomatic Hematuria, End-Stage Kidney Disease, and Undetected IgA Nephropathy at Age 18 y eFigure 1. State Transition Diagram of Mild IgA Nephropathy eFigure 2. State Transition Diagram of Severe IgA Nephropathy eFigure 3. State Transition Diagram of Asymptomatic Hematuria eFigure 4. State Transition Diagram of End-Stage Kidney Disease eFigure 5. State Transition Diagram of Undetected IgA Nephropathy at Age 18 y eAppendix 4. Trees From States of Non IgA Nephropathy, Mild or Severe IgA Nephropathy, Asymptomatic Hematuria, and IgA Nephropathy Undetected Before Age 18 y in Screening Strategy eFigure 6. Tree From No IgA Nephropathy State in Screening Strategy eFigure 7. Tree From Mild IgA Nephropathy State in Screening Strategy eFigure 8. Tree From Severe IgA Nephropathy State in Screening Strategy eFigure 9. Tree of Asymptomatic Hematuria State in Screening Strategy eFigure 10. Tree of Undetected IgA Nephropathy Before Age 18 y State in Screening Strategy eAppendix 5. Parameters eTable 1. Model Probability Parameters of Cost Effectiveness Analysis for School Urinary Screening eTable 2. Costs of Cost Effectiveness Analysis for School Urinary Screening eTable 3. Utility of Cost Effectiveness Analysis for School Urinary Screening eAppendix 6. Results of Scenario Analysis for Cost-Effectiveness of School Urinary Screening eTable 4. Analysis Results for Scenario 1 of Cost Effectiveness Analysis for School Urinary Screening eTable 5. Analysis Results for Scenario 2 of Cost Effectiveness Analysis for School Urinary Screening eAppendix 7. Probabilistic Sensitivity Analysis eFigure 11. Incremental Cost-Effectiveness Scatterplot eReferences. [file jamanetwopen-e2356412-s001.pdf]

Supplemental Online Content

Honda K, Akune Y, Goto R. Cost-effectiveness of school urinary screening for early detection of IgA nephropathy in Japan. *JAMA Netw Open*. 2024;7(2):e2356412. doi:10.1001/jamanetworkopen.2023.56412

eAppendix 1. Definitions of Mild and Severe IgA Nephropathy
eAppendix 2. Treatment of IgA Nephropathy
eAppendix 3. State Transition Diagram of Mild and Severe IgA Nephropathy, Asymptomatic Hematuria, End-Stage Kidney Disease, and Undetected IgA Nephropathy at Age 18 y
eFigure 1. State Transition Diagram of Mild IgA Nephropathy
eFigure 2. State Transition Diagram of Severe IgA Nephropathy
eFigure 3. State Transition Diagram of Asymptomatic Hematuria
eFigure 4. State Transition Diagram of End-Stage Kidney Disease
eFigure 5. State Transition Diagram of Undetected IgA Nephropathy at Age 18 y
eAppendix 4. Trees From States of Non IgA Nephropathy, Mild or Severe IgA Nephropathy, Asymptomatic Hematuria, and IgA Nephropathy Undetected Before Age 18 y in Screening Strategy
eFigure 6. Tree From No IgA Nephropathy State in Screening Strategy
eFigure 7. Tree From Mild IgA Nephropathy State in Screening Strategy
eFigure 8. Tree From Severe IgA Nephropathy State in Screening Strategy
eFigure 9. Tree of Asymptomatic Hematuria State in Screening Strategy
eFigure 10. Tree of Undetected IgA Nephropathy Before Age 18 y State in Screening Strategy
eAppendix 5. Parameters
eTable 1. Model Probability Parameters of Cost Effectiveness Analysis for School Urinary Screening
eTable 2. Costs of Cost Effectiveness Analysis for School Urinary Screening
eTable 3. Utility of Cost Effectiveness Analysis for School Urinary Screening
eAppendix 6. Results of Scenario Analysis for Cost-Effectiveness of School Urinary Screening
eTable 4. Analysis Results for Scenario 1 of Cost Effectiveness Analysis for School Urinary Screening
eTable 5. Analysis Results for Scenario 2 of Cost Effectiveness Analysis for School Urinary Screening
eAppendix 7. Probabilistic Sensitivity Analysis

© 2024 Honda K et al. *JAMA Network Open.*

eFigure 11. Incremental Cost-Effectiveness Scatterplot
eReferences.

This supplemental material has been provided by the authors to give readers additional information about their work.

1. Definitions of mild/severe IgA nephropathy

"Mild IgAN": IgAN with mild proteinuria and normal renal function with focal mesangial proliferation by renal biopsy. The clinical practice guidelines for pediatric IgA nephropathy in Japan[1] provide more detailed information on this classification, as follows: mild proteinuria (early morning protein/creatinine ratio less than 1.0), normal renal function (eGFR greater than 90 mL/min/1.73 m^2), and histopathological findings of less than 80% of glomeruli with mesangial cell proliferation, crescent formation, adhesion, or sclerotic glomeruli with crescentic formation are less than 30% of the total glomeruli.

"Severe IgAN": refers to cases of IgAN that do not meet the criteria for mild IgAN. Patients with rapidly progressive nephritis syndrome are not included in this category. The clinical practice guidelines for pediatric IgAN nephropathy in Japan[1] defines severe IgAN as follows: severe proteinuria (early morning protein/creatinine ratio > 1.0), impaired renal function (eGFR < 90 mL/min/1.73 m^2), histopathological findings of mesangial cell proliferation, crescent formation, adhesions, or sclerotic lesions in 80% or more of the glomeruli, or crescent formation in 30% or more of the glomeruli with mesangial cell proliferation, crescentic formation, adhesion, or sclerosing lesions.

2. Treatment of IgAN

For mild IgAN: treatment with angiotensin-converting enzyme inhibitor for 2 years (lisinopril, 5 mg/day for 6 months and 20 mg/day for the remaining 18 months).

For severe IgAN: combination therapy for 2 years (oral prednisolone: 60 mg/day for 4 weeks, 60 mg every other day for 4 weeks, 45 mg every other day for 4 weeks, 30 mg every other day for 9 months, and 15 mg every other day for the remaining 12 months; mizoribine 150 mg/day for 2 years; lisinopril 5 mg/day for 6 months and 20 mg/day for the remaining 18 months).

Both were performed in accordance with the clinical practice guidelines for pediatric IgA nephropathy[1]. Drug doses were calculated assuming a patient weight of 30 kg, based on guideline recommendations.

3. State transition diagram of mild IgAN, severe IgAN, Asymptomatic hematuria, end-stage renal disease, and undetected IgAN at age 18.

For mild IgAN, renal biopsy was sometimes not performed; instead, the patient had a follow-up check-up later (eFigure 1). For severe IgAN, renal biopsy was performed in all cases once detected (eFigure 2). Spontaneous remission could occur in mild IgAN but not in severe IgAN. After spontaneous remission in mild IgAN and after being detected in ASH, they were returned to the "non-IgAN" state after 5 years of routine check-ups (eFigure 1 and 3). Patients with dialysis continued dialysis until transplantation, but patients with PD were transferred to transplantation or HD after up to five years (eFigure 4).

© 2024 Honda K et al. *JAMA Network Open.*

IgAN: IgA nephropathy; ASH: asymptomatic hematuria; ESRD: end-stage renal disease; RBx: renal biopsy; PD: peritoneal dialysis; HD: hemodialysis; RTx: renal transplantation.

eFigure 1. State transition diagram of mild IgAN.

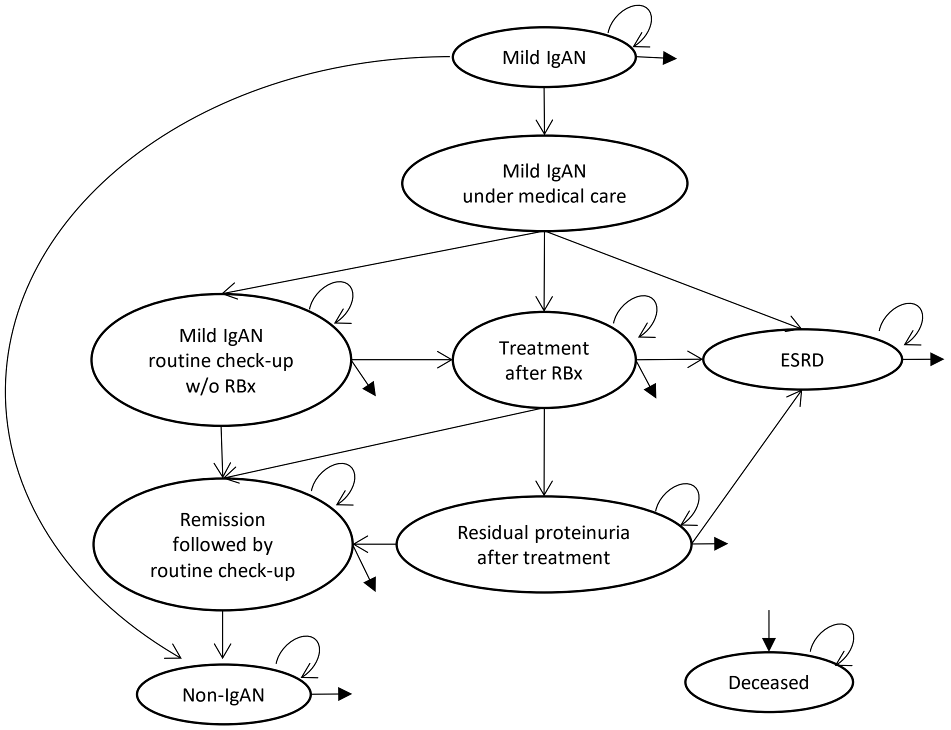

eFigure 2. State transition diagram of severe IgAN.

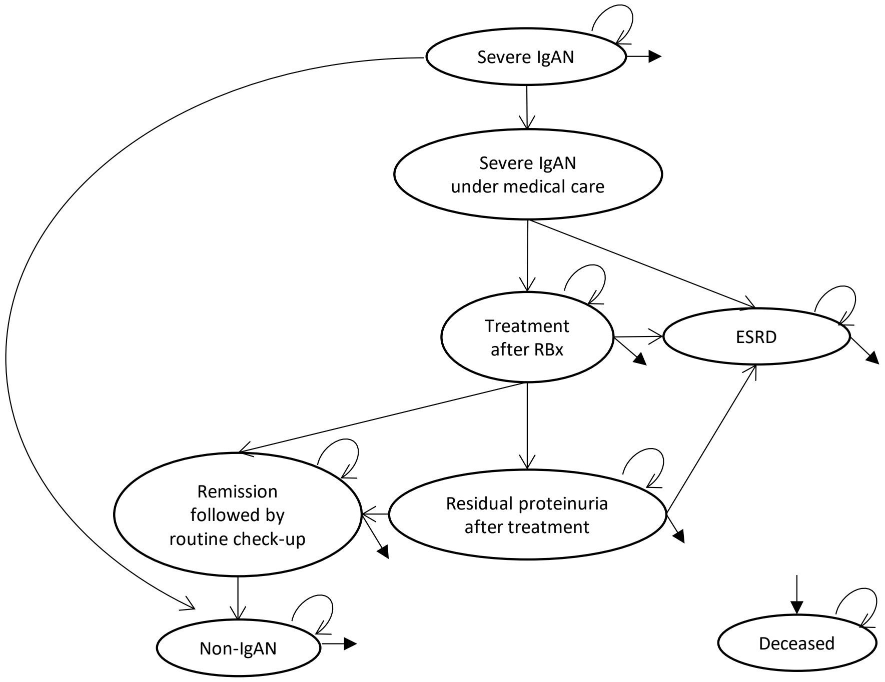

© 2024 Honda K et al. *JAMA Network Open.*

eFigure 3. State transition diagram of asymptomatic hematuria.

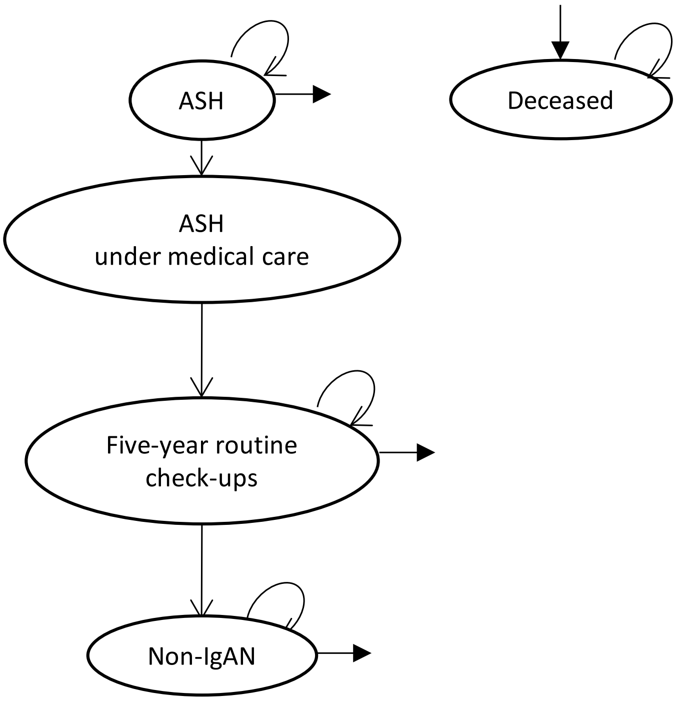

eFigure 4. State transition diagram of end-stage renal disease.

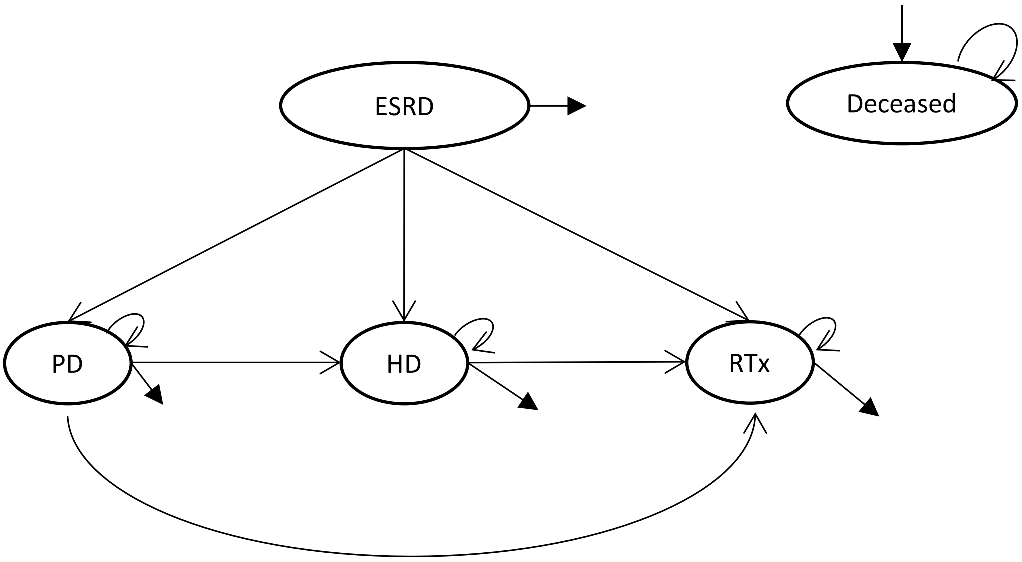

© 2024 Honda K et al. *JAMA Network Open.*

eFigure 5. State transition diagram of undetected IgAN at age 18.

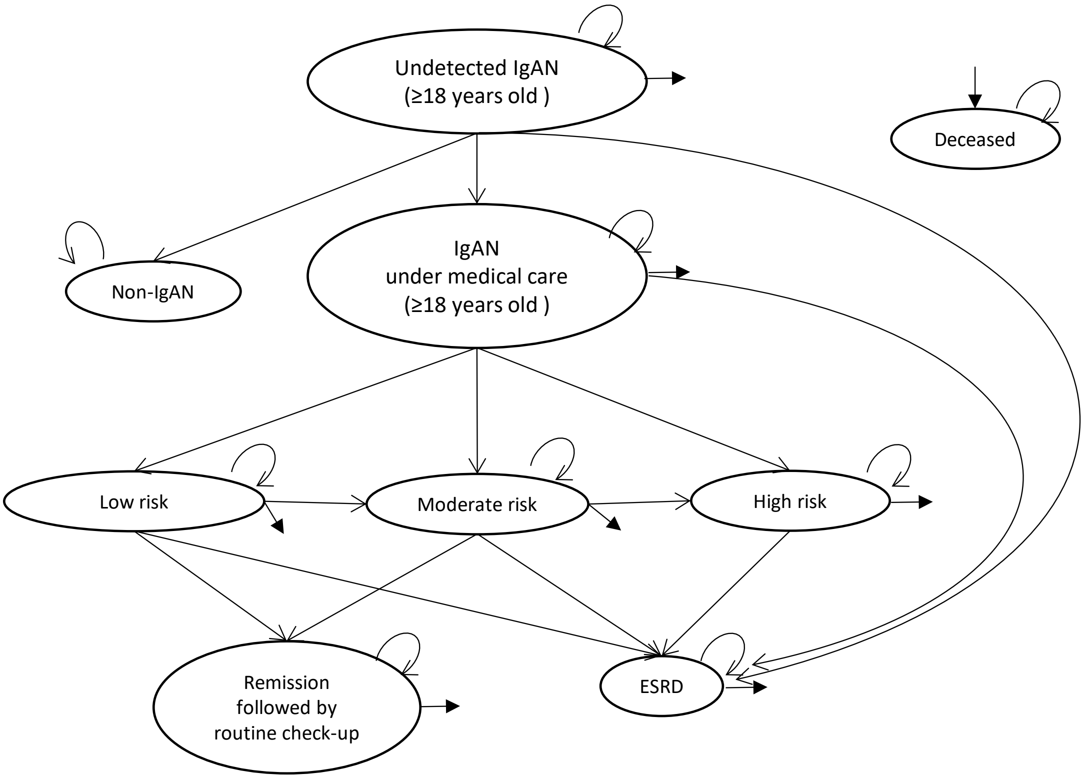

4. **Trees from states of "non-IgAN", "mild/severe IgAN", "ASH" and "IgAN undetected before age 18" in the screening strategy**

IgAN: IgA nephropathy; ESRD: end-stage renal disease; RBx: renal biopsy, ASH: asymptomatic hematuria

eFigure 6. Tree from "non-IgAN" state in the screening strategy.

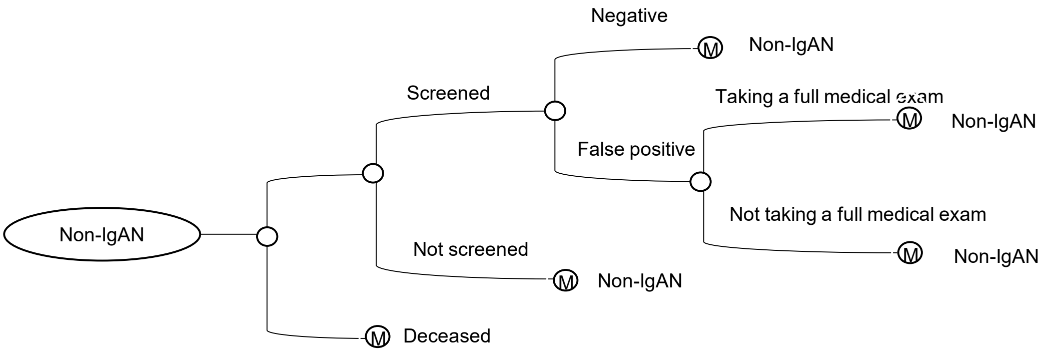

© 2024 Honda K et al. *JAMA Network Open.*

eFigure 7. Tree from "mild IgAN" state in the screening strategy.

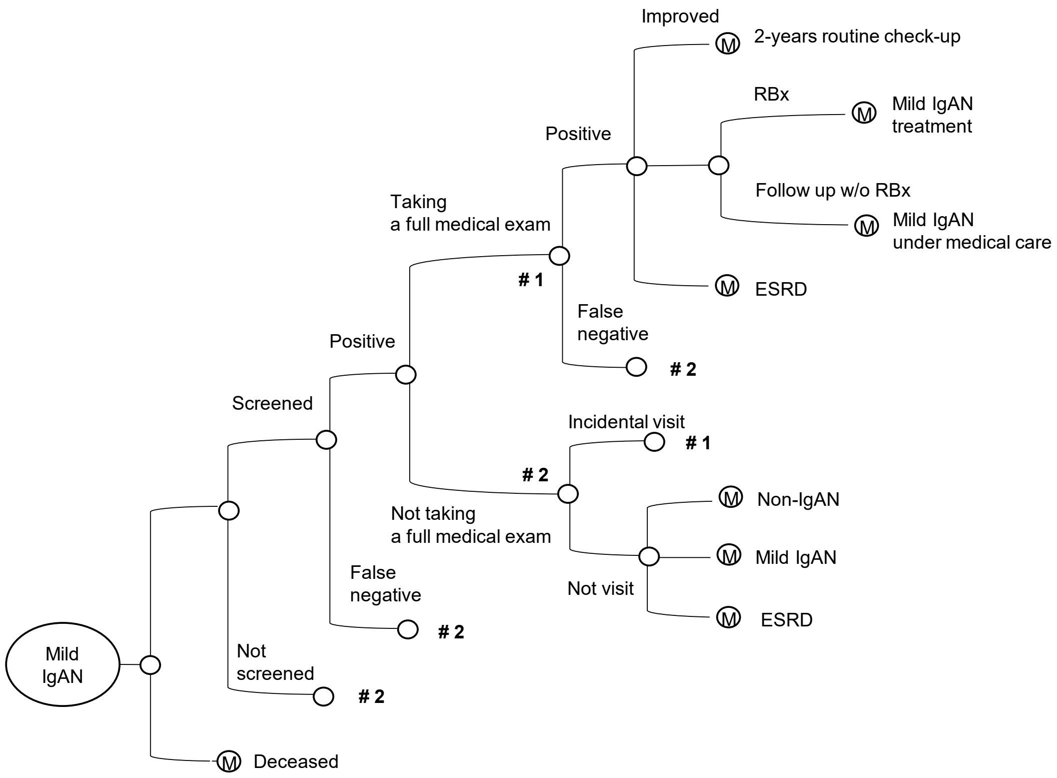

eFigure 8. Tree from "severe IgAN" state in the screening strategy.

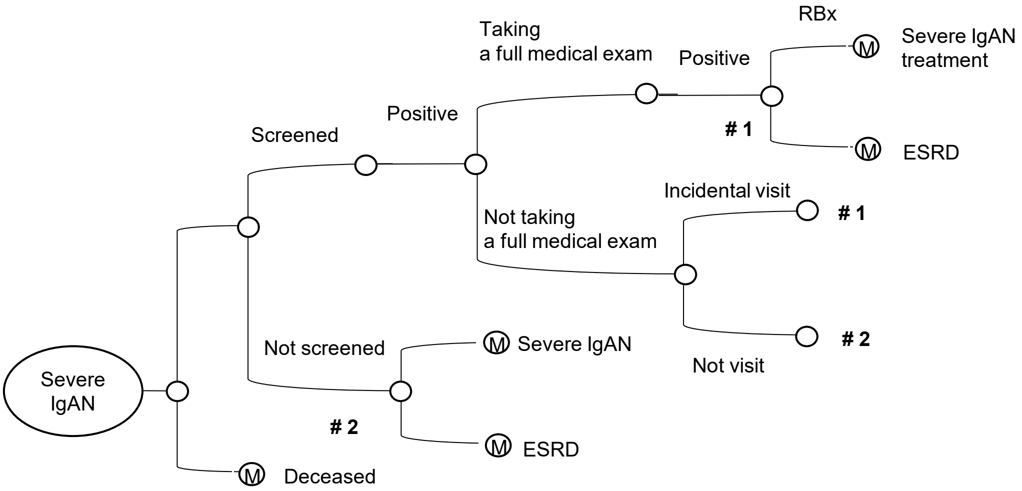

© 2024 Honda K et al. *JAMA Network Open.*

eFigure 9. Tree of the “asymptomatic hematuria” state in the screening strategy.

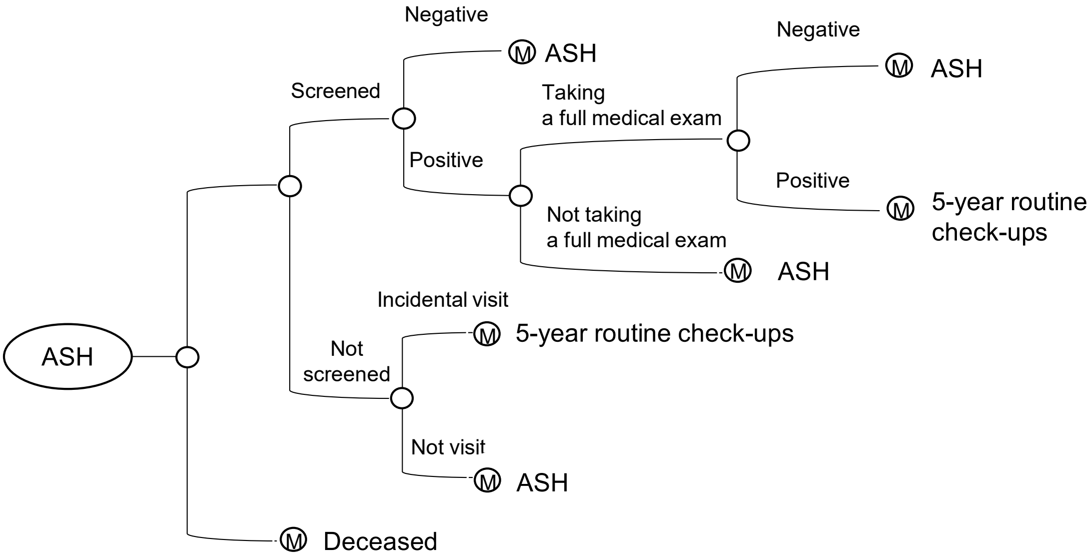

© 2024 Honda K et al. *JAMA Network Open.*

eFigure 10. Tree of "undetected IgAN before age 18" state in the screening strategy.

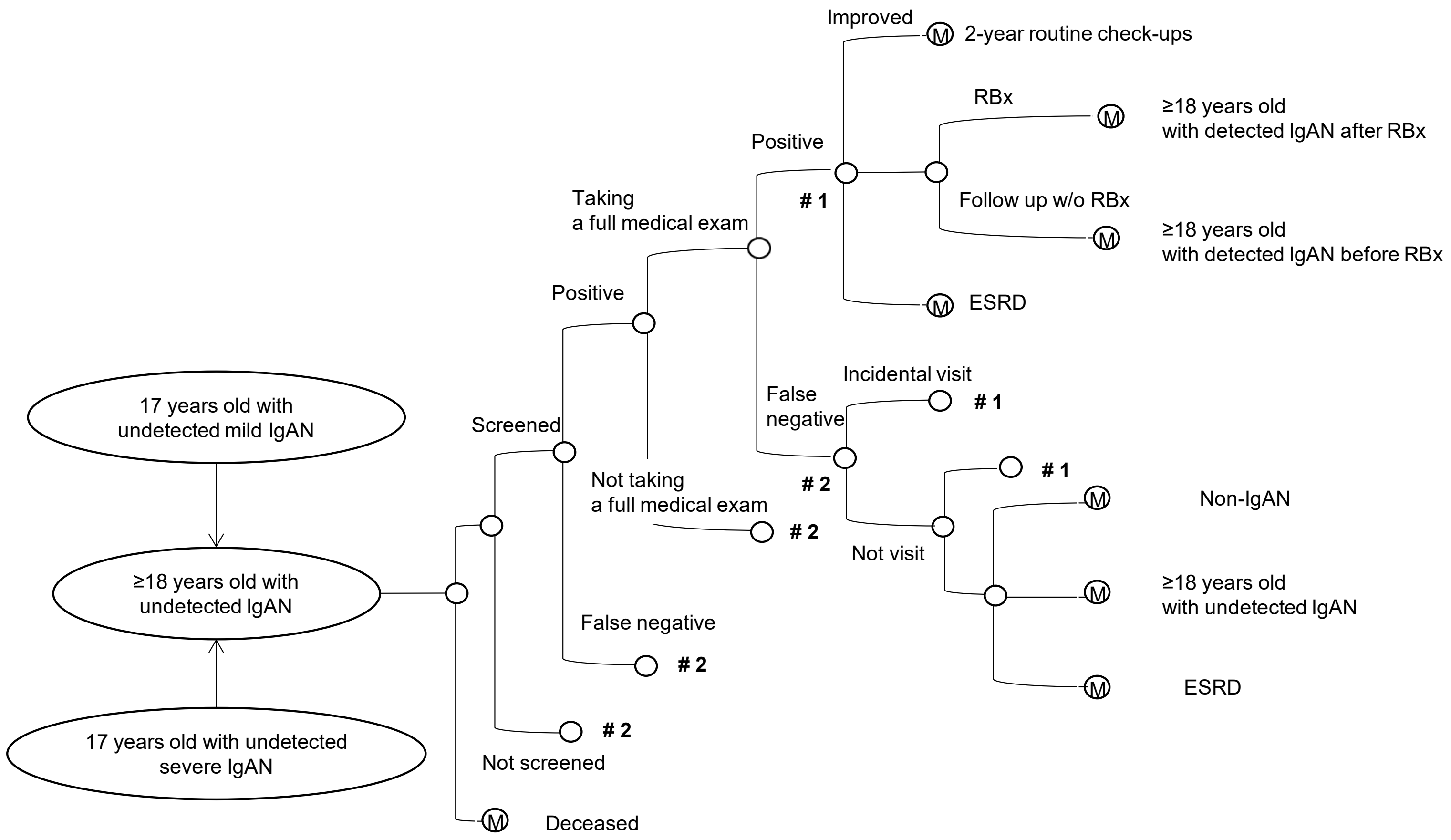

© 2024 Honda K et al. *JAMA Network Open.*

5. Parameters

eTable 1. Model Probability Parameters of the Cost Effectiveness Analysis for School Urinary Screening.

Parameter	Value	Low	High	Source #
Annual incidence of IgAN	0.000045	0.000045	0.000099	[2–4]
Proportion of severe IgAN	0.3	-50%	+50%	[2,5–7]
Proportion of low-risk patients in patients with IgAN (≥18 years)	0.36	-50%	+50%	[8]
Proportion of moderate-risk patients in patients with IgAN (≥18 years)	0.59	-50%	+50%	[8]
Proportion of high-risk patients in patients with IgAN (≥18 years)	0.05	-50%	+50%	[8]
Annual incidence of asymptomatic hematuria	0.00084	-50%	+50%	[9,10]
Annual probability of incidental visits in patients with IgAN	0.22	-50%	+50%	[2,3]
Annual probability of incidental visits in patients with ASH	0.1	-50%	+50%	*
Annual probability of incidental visits in patients with IgAN (≥18 years)	0.3	-50%	+50%	[11]
Annual probability of initiating treatment in patients with mild IgAN	0.2	-50%	+50%	*
Annual probability of RBx in patients with IgAN (≥18 years)	0.6	-50%	+50%	*
Annual probability of spontaneous remission among patients with mild IgAN	0.022	-50%	+50%	[12]
Annual probability of spontaneous remission in patients with IgAN (≥18 years)	0.003	-50%	+50%	[13]
Annual probability of progression to ESRD without treatment in patients with mild IgAN	0.0016	-50%	+50%	[5,7]
Annual probability of progression to ESRD without treatment in patients with severe IgAN	0.029	-50%	+50%	[7,14]
Annual probability of progression to ESRD in patients with IgAN (≥ 18 years) who are under medical care	0.016	-50%	+50%	[15]
Annual probability of progression to ESRD in patients with IgAN (≥ 18 years) not under medical care	0.022	-50%	+50%	[16,17]

© 2024 Honda K et al. *JAMA Network Open.*

Parameter	Value	Low	High	Source #
Annual probability of progression to ESRD in patients with low-risk IgAN (≥18years)	0.00093	-50%	+50%	[8]
Annual probability of progression to ESRD in patients with moderate-risk IgAN (≥18 years)	0.0059	-50%	+50%	[8]
Annual probability of progression to ESRD in patients with high-risk IgAN (≥18 years)	0.13	-50%	+50%	[8]
Proportion of remission at the end of treatment in patients with mild IgAN	0.89	-20%	+20%	[18]
Proportion of remission at the end of treatment in patients with severe IgAN	0.76	-20%	+20%	[19]
Annual probability of remission after residual urinary protein at the end of treatment in patients with mild IgAN	0.2	-50%	+50%	[a]
Annual probability of progression to ESRD after residual urinary protein at the end of treatment in patients with mild IgAN	0	0	0.0016	[14]
Annual probability of progression to ESRD after residual urinary protein at the end of treatment in patients with severe IgAN	0.012	-50%	+50%	[14]
Proportion of PD initiated after ESRD progression (<20 years)	0.62	-50%	+50%	[20]
Proportion of PD initiated after ESRD progression (≥20 years)	0.06	-50%	+50%	[21,22]
Proportion of HD initiated after ESRD progression (<20 years)	0.16	-50%	+50%	[20]
Proportion of HD initiated after ESRD progression (≥20 years)	0.9	-50%	+50%	[21,22]
Proportion of RTx implementation after ESRD progression (<20 years)	0.22	-50%	+50%	[20]
Proportion of RTx implementation after ESRD progression (≥20 years)	0.04	-50%	+50%	[21]
Probability of PD being continued for the next year among PD patients	0.8	-50%	1.0	[23]
Annual probability of RTx in PD/HD patients	0.14	-50%	+50%	[24]
Annual probability of progression to ESRD in patients with RTx (<20 years)	0.011	-50%	+50%	[25]
Annual probability of progression to ESRD in patients with RTx (≥20 years)	0.023	-50%	+50%	[21]

© 2024 Honda K et al. *JAMA Network Open.*

Parameter	Value	Low	High	Source #
Annual mortality in patients with dialysis (<20 years)	0.011	-50%	+50%	[20]
Annual mortality in patients with dialysis (≥20 years)	0.094	-50%	+50%	[22]
Annual mortality in patients with RTx (<20 years)	0.0021	-50%	+50%	[25]
Annual mortality in patients with RTx (≥20 years)	0.0098	-50%	+50%	[21]
Annual mortality in healthy, ASH, and IgAN population (without dialysis/RTx)	[b]	[c]		[26]
School urinary screening rate	0.98	[c]		[9]
Health check-up rate (≥19 years)	0.7	-50%	+50%	[27]
Probability of receiving a full medical examination (<18 years)	0.7	-50%	1.0	[2,9]
Probability of receiving a full medical examination (≥18 years)	0.4	-50%	+50%	[28]
False positive rate for school urine screening	0.0047	-50%	+50%	[2–4]
Sensitivity of school urine screening for IgAN	0.8	0.6	1.0	[a]
Sensitivity of school urine screening for ASH	0.8	0.6	1.0	[a]

[a] Author's assumption.
[b] Adapted mortality rates for each age group.
[c] Sensitivity analysis was not performed.
IgAN: IgA nephropathy; ASH: asymptomatic hematuria; RBx: renal biopsy; ESRD: end-stage renal disease; PD: peritoneal dialysis; HD: hemodialysis; RRT: renal replacement therapy; RTx: renal transplantation.

© 2024 Honda K et al. *JAMA Network Open.*

eTable 2. Costs of Cost Effectiveness Analysis for School Urinary Screening.

Parameter	Value (¥)	Low	High	Source
Screening	200	100	300	[29]
Detailed examination	18,940	-50%	+50%	[a]
RBx (<18 years)	276,388	-50%	+50%	[a]
Treatment of ACEI (1st year; <18 years)	200,610	-50%	+50%	[a]
Treatment of ACEI (2nd year; <18 years)	225,270	-50%	+50%	[a]
Treatment of combination therapy (1st year; <18 years)	420,234	-50%	+50%	[a]
Treatment of combination therapy (2nd year; <18 years)	441,351	-50%	+50%	[a]
Management of low-risk IgAN (annual, 1st year; ≥18 years)	318,502	-50%	+50%	[30]
Management of low-risk IgAN (annual, 2nd year and thereafter; ≥18 years)	25,682	-50%	+50%	[30]
Management of moderate-risk IgAN (annual, 1st year; ≥18 years)	914,744	-50%	+50%	[30]
Management of moderate-risk IgAN (annual, 2nd year, and thereafter; ≥18 years)	59,938	-50%	+50%	[30]
Management of high-risk IgAN (annual, 1st year; ≥18 years)	928,348	-50%	+50%	[30]
Management of high-risk IgAN (annual, 2nd year, and thereafter; ≥18 years)	122,444	-50%	+50%	[30]
Annual hospital visits for routine urine check-ups (<18 years)	7,020	-50%	+50%	[a]
Annual hospital visits among patients with mild IgAN (<18 years)	12,660	-50%	+50%	[a]
Annual hospital visits among patients with IgAN with residual proteinuria (without remission) after treatment (<18 years)	30,254	-50%	+50%	[a]

© 2024 Honda K et al. *JAMA Network Open.*

Parameter	Value	Low	High	Source #
HD (annual)	4,814,400	-50%	+50%	[31]
PD (annual)	6,314,400	-50%	+50%	[31]
RTx (annual, 1st year)	6,978,600	-50%	+50%	[31]
RTx (annual, 2nd year)	1,941,000	-50%	+50%	[31]
RTx (annual, 3rd year)	1,667,000	-50%	+50%	[31]
RTx (annual, 4th year)	1,683,000	-50%	+50%	[31]
RTx (annual, 5th year, and thereafter)	1,479,000	-50%	+50%	[31]

[a] Set according to the national medical care fee schedule and recommendations of expert committee/expert opinion.

ACEI: angiotensin-converting enzyme inhibitor; IgAN: IgA nephropathy; RBx: renal biopsy; PD: peritoneal dialysis; HD: hemodialysis; RTx: renal transplantation.

© 2024 Honda K et al. *JAMA Network Open.*

eTable 3. Utility of Cost Effectiveness Analysis for School Urinary Screening.

Health state	Value	Low	High	Source
Healthy	1.0	[a]		[b]
IgAN treatment	1.0	0.9	1.0	[b]
PD/HD (<20 years)	0.61	-20%	+20%	[32]
PD/HD (≥20 years)	0.75	-20%	+20%	[33]
RTx (<20 years)	0.76	-20%	+20%	[32]
RTx (≥20 years)	0.89	0.71	1.0	[34]

[a] Sensitivity analysis was not performed.
[b] Author's assumption.
PD: Peritoneal dialysis, HD: Hemodialysis, RTx: renal transplantation.

© 2024 Honda K et al. *JAMA Network Open.*

6. Results of Scenario Analysis for Cost-Effectiveness of School Urinary Screening

eTable4. Analysis Results for Scenario 1 of Cost Effectiveness Analysis for School Urinary Screening.

Starting age of screening	Cost (¥)	Incremental Cost (¥)	QALY	Incremental QALY	ICER	Number of patients with IgAN undetected before age 18[a]	Number of patients with ESRD[b] (lifetime)
No screening	8,338	NA	39.45001	NA	NA	135.5	60.3
10 years old	9,078	740	39.45036	0.00035	2,114,612	27.4	33.8
11 years old	8,890	551	39.45034	0.00034	1,635,594	27.4	34.6
12 years old	8,708	370	39.45033	0.00032	1,145,390	27.6	35.5
13 years old	8,535	196	39.45031	0.00031	638,493	28.2	36.5
14 years old	8,370	32	39.45030	0.00029	111,287	29.5	37.7
15 years old	8,223	-115	39.45027	0.00027	dominant	33.0	39.4
16 years old	8,113	-225	39.45024	0.00023	dominant	42.0	42.0
17 years old	8,096	-242	39.45017	0.00016	dominant	66.1	47.4

QALY: quality-adjusted life-years, ICER: incremental cost-effectiveness ratios, IgAN: IgA nephropathy, ESRD: end-stage renal disease.

[a]Number of patients per million

[b]Cumulative number of patients per million over the time horizon

© 2024 Honda K et al. *JAMA Network Open.*

eTable5. Analysis Results for Scenario 2 of Cost Effectiveness Analysis for School Urinary Screening.

Screening frequency	Cost (¥)	Incremental Cost (¥)	QALY	Incremental QALY	ICER	Number of patients with IgAN* undetected before age 18	Number of patients with ESRD* (lifetime)
No screening	8,338	NA	39.45001	NA	NA	135.5	60.3
Every two years (starting at age 6)	8,930	592	39.45027	0.00026	2,252,613	65.2	40.8
Every two years (starting at age 7)	8,761	423	39.45032	0.00032	1,337,699	41.4	36.6
Every three years (starting at age 6)	8,694	356	39.45019	0.00019	1,887,875	88.0	46.4
Every three years (starting at age 8)	8,431	93	39.45028	0.00027	338,734	49.7	39.6
Three times (at ages 11, 14, 17)	8,263	-75	39.45026	0.00025	dominant	50.1	40.9
Once (at age 14)	8,287	-51	39.45010	0.00009	dominant	106.5	53.2

QALY: quality-adjusted life-years, ICER: incremental cost-effectiveness ratios, IgAN: IgA nephropathy, ESRD: end-stage renal disease.

[a]Number of patients per million

[b]Cumulative number of patients per million over the time horizon

© 2024 Honda K et al. *JAMA Network Open.*

7. Probabilistic sensitivity analysis

eFigure 11. Incremental cost-effectiveness scatterplot.

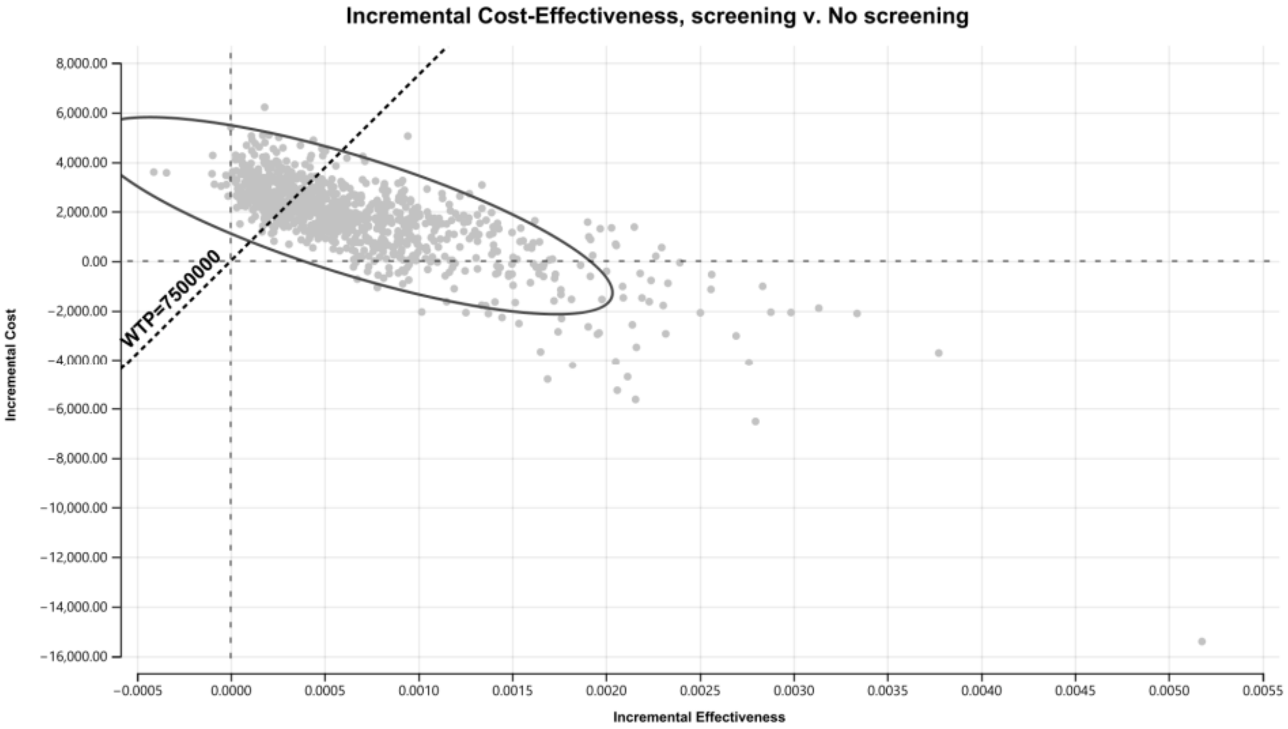

WTP: willingness to pay

The diagonal dashed line represents a ¥ 750,000 ($70,093 US) per quality-adjusted life-year threshold. Points falling below this line represent simulations that were cost-effective.

© 2024 Honda K et al. *JAMA Network Open.*

8. eReference

1. The Japanese Society for Pediatric Nephrology. Clinical Practice Guidelines for IgA Nephropathy 2020 [Translated from Japanese.]. Shindan-to-Chiryo Sha Inc; 2020

2. Utsunomiya Y, Koda T, Kado T, et al. Incidence of pediatric IgA nephropathy. Pediatr Nephrol. 2003;18(6):511-515. doi:10.1007/s00467-003-1127-z

3. Ikezumi Y, Suzuki T, Karasawa T, Uchiyama M. Epidemiological surveys of pediatric IgA nephropathy based on a urinalysis screening program in Niigata-city and a surveillance network for pediatric nephritic syndrome in Niigata prefecture. Jpn Soc Pediatr Nephrol. 2008;21(2):110-115. doi:10.3165/jjpn.21.110

4. Shibano T, Takagi N, Maekawa K, et al. Epidemiological survey and clinical investigation of pediatric IgA nephropathy. Clin Exp Nephrol. 2016;20(1):111-117. doi:10.1007/s10157-015-1129-8

5. Higa A, Shima Y, Hama T, et al. Long-term outcome of childhood IgA nephropathy with minimal proteinuria. Pediatr Nephrol. 2015;30(12):2121-2127. doi:10.1007/s00467-015-3176-5

6. Yoshikawa N, Ito H, Nakamura H. Prognostic indicators in childhood IgA nephropathy. Nephron. 1992;60(1):60-67. doi:10.1159/000186706

7. Yata N, Nakanishi K, Shima Y, et al. Improved renal survival in Japanese children with IgA nephropathy. Pediatr Nephrol. 2008;23:905-912. doi:10.1007/s00467-007-0726-5

8. Katafuchi R, Ninomiya T, Nagata M, Mitsuiki K, Hirakata H. Validation study of oxford classification of IgA nephropathy: the significance of extracapillary proliferation. Clin J Am Soc Nephrol. 2011;6(12):2806-2813. doi:10.2215/CJN.02890311

9. Matsumura C. School urinary screening in Chiba City [Translated from Japanese.]. J Pediatr Pract. 2013;66(4):623-629.

10. Japan Society of School Health. Report on Survey Project on Healthcare in School Life, 2013 [Translated from Japanese]

11. Clinical Guidelines for IgA Nephropathy 2020 Advisory Committee. Evidence-Based Clinical Practice Guidelines for Nephrotic Syndrome [Translated from Japanese]. Tokyo Igakusha Inc; 2020.

12. Shima Y, Nakanishi K, Hama T, et al. Spontaneous remission in children with IgA nephropathy. Pediatr Nephrol. 2013;28(1):71-76. doi:10.1007/s00467-012-2294-6

13. Costa RS, Droz D, Noel LH. Long-standing spontaneous clinical remission and glomerular improvement in primary IgA nephropathy (Berger's disease). Am J Nephrol. 1987;7(6):440-444. doi:10.1159/000167516

14. Kamei K, Nakanishi K, Ito S, et al. Long-term results of a randomized controlled trial in childhood IgA nephropathy. Clin J Am Soc Nephrol. 2011;6(6):1301-1307. doi:10.2215/cjn.08630910

15. Goto M, Wakai K, Kawamura T, Ando M, Endoh M, Tomino Y. A scoring system to predict renal outcome in IgA nephropathy: a nationwide 10-year prospective cohort study. Nephrol Dial Transplant. 2009;24(10):3068-3074. doi:10.1093/ndt/gfp273

16. D'Amico G. Natural history of idiopathic IgA nephropathy: Role of clinical and histological prognostic factors. Am J Kidney Dis. 2000;36(2):227-237. doi:10.1053/ajkd.2000.8966

© 2024 Honda K et al. *JAMA Network Open.*

17. Kusumoto Y, Takebayashi S, Taguchi T, Harada T, Naito S. Long-term prognosis and prognostic indices of IgA nephropathy in juvenile and in adult Japanese. Clin Nephrol. 1987;28(3):118-124.

18. Shima Y, Nakanishi K, Sako M, et al. Lisinopril versus lisinopril and losartan for mild childhood IgA nephropathy: a randomized controlled trial (JSKDC01 study). Pediatr Nephrol. 2019;34(5):837-846. doi:10.1007/s00467-018-4099-8

19. Shima Y, Nakanishi K, Kaku Y, et al. Combination therapy with or without warfarin and dipyridamole for severe childhood IgA nephropathy: an RCT. Pediatr Nephrol. 2018;33(11):2103-2112. doi:10.1007/s00467-018-4011-6

20. Hattori M, Sako M, Kaneko T, et al. End-stage renal disease in Japanese children: a nationwide survey during 2006–2011. Clin Exp Nephrol. 2015;19(5):933-938. doi:10.1007/s10157-014-1077-8

21. The Japan Society for Transplantation. Fact Book 2021 on Organ Transplantation in Japan. 2021. Available at: http://www.asas.or.jp/jst/pdf/factbook/factbook2021.pdf. Accessed July 15, 2022.

22. Hanafusa N, Abe M, Joki N, et al. わが国の慢性透析療法の現況 2020 Annual Dialysis Data Report, JSDT Renal Data Registry. 透析会誌 J Jpn Soc Dial Ther. 2021;54(12):611-657.

23. Kamei K, Sato M, Murakoshi M, et al. Primary disease, age, and outcomes of 70 patients who were started on peritoneal dialysis at our center [Translated from Japanese.]. J Jpn Soc Dial Ther. 2020;53(7):411-417.

24. Hattori M, Sako M, Kaneko T, et al. Report on epidemiological survey of pediatric end-stage renal failure patients in Japan: with special reference to dialysis therapy [Translated from Japanese.]. J Jpn Soc Dial Ther Jpn. 2014;47(2):167-174. doi:10.4009/jsdt.47.167

25. Hattori M, Mieno M, Aikawa A, et al. Demographic data and outcome of pediatric kidney transplantation in Japan [Translated from Japanese.]. Journal Jpn Soc Clin Ren Transplant. 2016;4(2):301-312.

26. Statistics Bureau, Ministry of Internal Affairs and Communications. Statistics Bureau of Japan. Published 2021. Accessed December 6, 2021. https://www.stat.go.jp/english/index.html

27. Household Statistics Office, Director-General for Statistics and Information Policy. Health of Household Members. Comprehensive Survey of Living Conditions. Accessed May 25, 2023. https://www.mhlw.go.jp/english/database/db-hss/cslc-report2019.html

28. Kondo M, Yamagata K, Hoshi SL, et al. Cost-effectiveness of chronic kidney disease mass screening test in Japan. Clin Exp Nephrol. 2012;16(2):279-291. doi:10.1007/s10157-011-0567-1

29. Murakami M, Masami T. Achievements and problems with the current mass urinalysis system in Japan [Translated from Japanese.]. Japanese J Pediatr Med. 2003;35(5):853-856.

30. Okubo R, Hoshi SL, Kimura T, et al. Cost-effectiveness of mass screening for dipstick hematuria in Japan. Clin Exp Nephrol. 2022;26(5):398-412. doi:10.1007/s10157-021-02170-0

31. Uchida J, Nakatani T. Renal replacement therapy and economic efficiency [Translated from Japanese.]. Kidney Dial. 2011;71(3):402–407. 32.

32. Francis A, Didsbury MS, Van Zwieten A, et al. Quality of life of children and adolescents with chronic kidney disease: A cross-sectional study. Arch Dis Child. 2019;104(2):134-140. doi:10.1136/archdischild-2018-314934

© 2024 Honda K et al. *JAMA Network Open.*

33. Noto S, Miyazaki M, Takeuchi H, Saito S. Relationship between hemodialysis and health-related quality of life: a cross-sectional study of diagnosis and duration of hemodialysis. Ren Replace Ther. 2021;7(1):1-8. doi:10.1186/s41100-021-00382-4

34. Hiragi S, Goto R, Tanaka Y, et al. Estimating the Net Utility Gains Among Donors and Recipients of Adult Living Donor Kidney Transplant. Transplant Proc. 2019;51(3):676-683. doi:10.1016/j.transproceed.2019.01.049

© 2024 Honda K et al. *JAMA Network Open.*
