# Supplementary material for: Cost-Effectiveness of School Urinary Screening for Early Detection of IgA Nephropathy in Japan
Source: JAMA Netw Open. 2024 Feb 16;7(2):e2356412. doi: 10.1001/jamanetworkopen.2023.56412 (PMC10873767; doi:10.1001/jamanetworkopen.2023.56412)
Supplement: Supplement 2. — Data Sharing Statement [file jamanetwopen-e2356412-s002.pdf]

Data Sharing Statement

Honda. Cost-Effectiveness of School Urinary Screening for Early Detection of IgA Nephropathy in Japan. *JAMA Netw Open*. Published online February 16, 2024. doi:10.1001/jamanetworkopen.2023.56412

Data
Data available: Yes
Data types: Other (please specify)
Additional Information: Computer simulation model(Treeage) **How to access data:** kimikohondamd@gmail.com
When available: With publication

Supporting Documents
Document types: None

Additional Information
Who can access the data: anyone requesting the data
Types of analyses: for any purpose or for a specified purpose
Mechanisms of data availability: after approval of a proposal
